# Preparing clinicians for practice: effectiveness and design of on-call simulation

**DOI:** 10.1186/s12909-024-05495-y

**Published:** 2024-06-05

**Authors:** Sebastian Priest, Lucy Wells, Hajnalka Huszka, Nick Tovell, Michael Okorie

**Affiliations:** 1 University Hospitals Sussex NHS Foundation Trust, Brighton, UK; 2 Brighton and Sussex Medical School, Brighton, UK

**Keywords:** On-call, Simulation, Medical education, Medical student, Foundation doctors

## Abstract

**Background:**

Final year medical students and postgraduate doctors regularly contend with feelings of under-preparedness when transitioning into new areas of clinical practice. This lack of confidence is most evident in the context of on-call work which frequently requires sound clinical prioritisation, rigorous decision making and the management of acutely unwell patients, often with reduced senior support and staffing. This has prompted the emergence of on-call simulation which seeks to enhance participant confidence in performing on-call tasks and facilitate the development of key clinical and non-technical skills. This narrative review examined the use of on-call simulation in medical student and newly qualified doctor cohorts, its effectiveness in achieving its stated outcomes and to identify novel areas for the development of existing models.

**Method:**

A search strategy was developed in conjunction with a specialist medical librarian. OVID Medline and Embase searches identified articles related to the use and design of on-call simulation in medical education with no restrictions placed upon date or language of publication. Key findings from articles were summarised to develop comprehensive themes for discussion.

**Results:**

Twenty Three unique publications were reviewed which unanimously reported that on-call simulation had a positive effect on self-reported participant confidence in performing on-call roles. Furthermore the value on-call simulation when used as an induction activity was also evident. However, there was limited evidence around improved patient and performance outcomes following simulation. It also remains resource intensive as an educational tool and there is a distinct absence of interprofessional education in current models.

**Conclusions:**

We concluded that on-call simulation must adopt an interprofessional educational approach, incorporating other clinical roles. Further studies are needed to characterise the impact on patient outcomes. It remains highly useful as a confidence-boosting induction activity, particularly in specialities where clinical exposure is limited. Virtual and tabletop simulation formats, could potentially address the resource burden of manikin-based models, particularly with ever growing demands on medical educators and the expansion of training posts.

**Supplementary Information:**

The online version contains supplementary material available at 10.1186/s12909-024-05495-y.

## Background

The landscape of medical education has evolved significantly over the years, with a growing emphasis on emulating the realism of clinical practice through simulation training [1]. This approach facilitates experiential learning, giving individuals the opportunity to cultivate both technical and non-technical skills in a controlled environment [2]. Furthermore, in the current era of limited capacity of clinical staff, simulation has enabled learning to occur with minimal disruption to delivery of the clinical service [1, 2].

Despite this, existing evidence suggests that many final year medical students and newly qualified doctors frequently lack confidence and contend with feelings of under-preparedness when transitioning into clinical practice [3, 4]. This is most evident in the context of on-call work which includes the management of acutely unwell patients and clinical prioritisation - tasks which are viewed as particularly challenging [5]. Consequently, the emergent use of on-call simulation has not only enhanced participant confidence in performing on-call tasks but has also facilitated the development of key clinical and non-technical abilities [3, 6, 7].

On-call simulation sessions typically involve participants taking control of a pager device where they are contacted by facilitators requesting their assistance with a range of simulated clinically urgent and non-urgent tasks [8]. Tasks are designed to reflect the typical jobs a UK-based foundation doctor may be expected to complete while on-call. Tasks may range from reviewing a deteriorating patient to interpreting routine investigations and completing discharge documentation. The role of participants is to prioritise and perform these requests according to clinical necessity.

The proposed introduction of apprenticeships to confer a degree supporting the title “medical doctor” in the UK combined with increasing capacity pressures on traditional ward teaching formats will need to be addressed if the standards of training are to be maintained and developed [9]. Simulation may present an opportunity to mitigate the impact of ever-growing numbers of students, the pressures educators face to accommodate them and, the associated dilution of access to educational experiences and resultant erosion of quality. However, within simulation, further innovation is still required to address these capacity pressures. Tabletop methods, including low-fidelity card and board game exercises, have become an emergent practice in the field particularly in areas where logistical and resource constraints must be considered on a large scale [10, 11]. Their subsequent implementation may help address some of the issues outlined.

Three key aims will be explored throughout this review.

1. Examine the use of on-call simulation in medical student and newly qualified doctor cohorts
2. Evaluate the effectiveness of on-call simulation as an educational methodology in achieving its stated outcomes
3. Identify novel areas for the development of existing models

## Method

Medline and Embase searches were conducted using OVID following the development of a strategy in conjunction with a specialist medical librarian. The full strategy, including terms searched is available in appendix 1 and 2. The search placed no limit on the date of publication or language of publication. Titles were screened and duplicates removed in Endnote by the medical librarian.

Following this, full-text articles and abstracts were reviewed for relevance by a single reviewer. Publications were included if they addressed the topic of on-call simulation relating to either original research or review articles on the educational methodology in medical student or newly qualified doctor cohorts.. For the purposes of this review, newly qualified doctors relates to doctors in their first two years of clinical practice. Articles discussing the impact of other educational tools such as handbooks and preparatory courses on augmenting participant confidence in new clinical environments, but not relating to on-call work or on-call simulation, were excluded. The inclusion and exclusion criteria are formally documented in appendix 3.

Full-text articles were reviewed to extract the following data by a single investigator. The following elements were reviewed for analysis:

- Simulation Methodology (Single or multiple station simulation)
- Site and method of delivery including virtual/in-person simulation.
- Clinical specialty of delivery (General Medicine, Psychiatry etc.)
- Role of participants (Medical Students, Doctors)
- Outcome measures including subjective and objective assessments.
- Key remarks and learning points identified by authors relating to methodology design and delivery.

Following data extraction the above criteria were summarised in a narrative format for each publication by the single investigator. Summaries were reviewed by this reviewer to help identify and develop encompassing themes to prompt discussion on the effectiveness of on-call simulation, the extent of its use and novel areas for improvement.

## Results

**Fig. 1:**
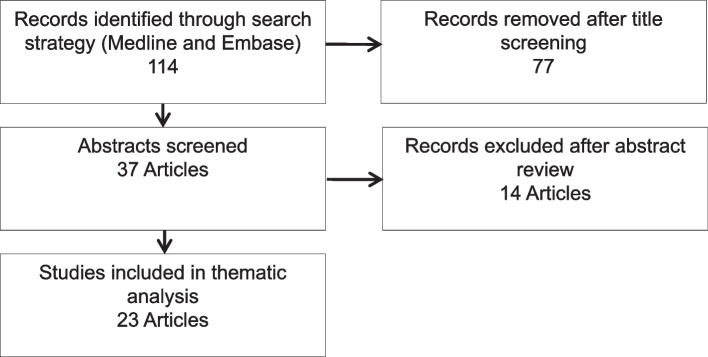
Flowchart illustrating study selection

The search was performed on the 18th August 2023 using OVID Medline and Embase, with the strategy and search numbers outlined in appendix 2. This yielded 37 results which included a combination of full-text articles and conference abstracts (Fig. 1) [12]. Fourteen results were excluded, and 23 articles were analysed within the scope of this literature review. All participants in the simulations reviewed were either medical students or newly qualified doctors.

### Key findings

All of the reviewed papers showed an improvement in participant feelings of self-reported confidence and preparedness in performing on-call tasks immediately following simulation. Self-reported confidence measures were collected immediately prior to and post the simulation activity in all but 1 of the 23 reviewed articles. Carpenter et al*.* demonstrated enhanced levels of participant confidence immediately following simulation amongst final year medical students [5]. However, at 6 month follow-up of participants who were now working as newly graduated doctors, the authors found that self-reported confidence in performing on-call tasks was lower than immediately following simulation despite further clinical experience. Debrief feedback and free-text questionnaires consistently reported positive comments in relation to the simulation experience [13–15].

A review of the narrative summaries for each article identified and developed the following four themes for discussion:

1. Critical aspects of simulation design
2. Limited assessment of objective performance outcomes
3. Effectiveness as an induction activity
4. On-call simulation remains resource intensive

### Critical aspects of simulation design

In order to identify novel areas for improvement the use and design of current on-call simulations was examined. A number of publications incorporated qualitative feedback into their evaluative process. This allowed study authors to reflect on the strengths and limitations of their model and enhance their simulations for future participants [16].

Udeaja et al. advised that the design of performed tasks was critical to the success of any simulation [6]. The aim was to provide participants with common scenarios that reflect the realism of clinical practice, allowing them to develop and reproduce critical skills in the workplace (Table 1). Carpenter et al. went further to suggest that stakeholder consultations should be conducted prior to simulation creation, involving students and newly qualified doctors to help identify common tasks that should be addressed and highlight key learning needs [5]. Nevertheless, the authors did not clarify what form these stakeholder consultations took. The use of educational adjuncts such as tutorials and lectures prior to simulation was also found to improve learning outcomes and reduce logistical difficulties associated with performing simulated tasks [5, 10, 17].

**Table 1:** Key findings on critical aspects of on-call simulation design

**Key findings**
Simulation tasks must reflect the realities of clinical practice
Stakeholder consultations can help inform task design
Pre-simulation tutorials can be useful adjuncts
Inclusion of high fidelity simulation tools can enhance realism
Ensuring on-call simulation is challenging can enhance realism

High fidelity tools in on-call simulation consistently received positive feedback across the reviewed literature as they were felt to enhance the realism of situations [9, 18]. Participants often praised the inclusion of scenarios utilising manikin models, professional actors and simulated patients.

A number of participants and facilitators also emphasised the importance of challenging participants in the on-call simulation environment [10]. This was achieved in several ways including the use of distraction tasks [6, 11]. These requests are often clinically non-urgent but are designed to test participants in their ability to prioritise effectively. Examples included the completion of discharge documentation and routine updates for next of kin. Likewise, the logistical challenges of completing tasks in-situ while navigating an unfamiliar environment received positive feedback [6]. This, alongside perceived time pressure [10], created by challenging trainees with a high volume of pager requests, were felt to enrich the psychological realism of simulation.

### Limited assessment of objective performance outcomes

Three publications reported improvements in assessments of participant performance following on-call simulation [3, 12, 19]. These assessments were either made on the judgement of facilitators observing participant performance during the simulation or by collating feedback from senior clinicians who worked with participants, post-simulation, in the clinical environment. It remains unclear how and when this was assessed in the latter approach [3, 18]. None of the reviewed articles examined any patient-related outcomes (Table 2).

**Table 2:** Key findings on the objective assessment of outcomes in on-call simulation

***Key findings***
Objective assessment of performance is limited in on-call simulation and reported by simulation facilitators
Objective assessment using scoring tools is focussed on participant performance in subsequent simulations
Assessment of patient related outcomes are absent in relation to on-call simulation

Only 1 of the 23 articles reviewed introduced an objective scoring system to document participant performance and their progress in subsequent simulations [12]. This simulation involved final year medical students holding a pager over a seven-week period during normal clinical placement activities. They were subsequently paged on four separate occasions to attend a simulated emergency scenario that they were expected to manage. Three members of the faculty then evaluated participant’s technical performance independently using the Objective Simulation Assessment Tool (OSAT) [20]. The marking criteria for the OSAT was derived from the standard ABCDE approach to emergency situations, with participants scoring positively for following the correct sequence of assessment and initiating appropriate management strategies.

### Effectiveness as an induction activity

Three of the studies reviewed had either incorporated on-call simulations into their induction programmes or advocated that such training be mandatory as part of their departmental introductions (Table 3) [5, 20, 21]. Authors highlighted that this was particularly useful in specialities where medical school exposure is limited [5].

**Table 3:** Key findings on the effectiveness of on-call as an induction activity

**Key findings**
On-call simulations are a useful induction activity
On-call simulations are particularly useful in clinical specialties where participants have limited previous exposure
A blended lecture-simulation approach remains superior to an induction lecture in isolation

Blamey et al. developed a simulation addressing common psychiatric on-call tasks for incoming foundation doctors at a mental health trust [7]. The programme consisted of a didactic lecture discussing common pager requests received by foundation doctors during their rotations. This was followed by a 2-h virtual on-call simulation where participants had to manage requests ranging from seclusion reviews to prescribing medications for acute psychiatric presentations. Self-reported confidence ratings collected immediately prior to and following simulation demonstrated that the simulation improved trainee confidence significantly when compared to the didactic lecture. The authors concluded that although the didactic teaching format was an important adjunct, they reaffirmed that the act of simulation was critical in helping participants transition their theoretical knowledge into practical knowledge [21].

### On-call simulation remains resource intensive

The challenge of delivering training through the COVID-19 pandemic led to innovative change and a move towards online-based simulation [5, 16, 22]. Four publications explored the substitution of in-situ training with a virtual experience [1, 5, 17, 23]. The feedback on virtual training remained consistent with in-situ simulation, with quantitative data demonstrating subjective improvements in confidence when managing on-call tasks (Table 4). Authors also reported that online simulation has the potential for greater scalability and accessibility with the use of automation [16].

**Table 4:** Key findings on resource use in on-call simulation

**Key findings**
Positive self-reported outcomes on virtual simulations are consistent with in-situ models
Virtual simulations have greater potential for scalability and accessibility

## Discussion

### Critical aspects of simulation design

Qualitative feedback of the examined articles often highlighted the importance of realism and that simulations reflected the clinical environment closely [4, 9, 18]. A number of studies achieved this by ensuring simulated tasks were designed carefully to reflect the true nature of on-call work [3, 8]. The importance of challenging participants through distraction tasks and high volumes of pager requests, alongside navigating unfamiliar clinical environments was also identified [6, 7]. However, it is important to consider that psychological burden and level of immersion should be chosen carefully; especially with on-call-naïve and medical student participants.

High fidelity techniques also received praise from participants as contributing to the realism of simulation [5, 18]. However, a review of combined high fidelity and low fidelity techniques in a mixed on-call and ward simulation did not suggest that low fidelity systems were inferior [5]. It could therefore be proposed that low fidelity simulation still performs the function of allowing participants to practice key clinical and non-technical skills including prioritisation, decision making and prescribing without the need for resource intensive adjuncts.

### Limited assessment of objective performance outcomes

There remains a paucity of objective assessments relating to both participant performance and patient-related outcomes in the context of on-call simulation. Although 3 studies did report positive developments in participant clinical performance following simulation, the feedback does not specify which competencies had been developed and how performance had improved [5, 17, 19]. It remains difficult quantifying improvements in clinical practice, particularly as a number of other factors including further clinical experience are likely to have an impact on performance. Comprehensive quantifiable assessments of subsequent clinical ability are therefore challenging to construct.

Watmough et al*.* exploration of OSAT scoring did establish a generalised improvement in performance scores in subsequent simulated scenarios, although interpretation was often limited by small sample sizes [17]. While this on-call simulation differs from the traditional on-call model, where trainees are allocated multiple tasks of varying priority in a set period, it does highlight a method through which gains in technical competency could be objectively assessed. Nevertheless, this does not address the gap in objective, quantified assessments of non-technical skills such as prioritisation and decision making following on-call simulation.

Ultimately, there remains a distinct lack of objective assessment in the majority of on-call simulation publications. This raises questions as to whether this form of simulation does lead to enhanced performance and development of non-technical skills. It can be argued that feelings of under-preparedness when working in the clinical environment have been the common catalyst necessitating the development of these simulations [3, 4]. Therefore, subjective ratings of improved confidence amongst participants are a meaningful metric in the evaluation of the benefit of on-call training [3, 5, 8]. Nonetheless, questions remain as to whether these benefits confer any long-lasting impact as outlined by Carpenter et al*.* and further investigation is required [5].

### Effectiveness as an induction activity

Effective inductions for doctors in training facilitate enhanced transitions into the working environment and can build confidence when working in complex, unfamiliar situations [24]. A GMC case study of the Portsmouth Department of Critical Care [25] demonstrated how simulation plays a key role in facilitating effective inductions where clinical scenarios, hospital logistics and human factors are addressed. Although this case study related to acute patient simulation rather an on-call simulation with an array of different tasks, it reinforces the potential of simulation as an induction activity.

However, in departmental inductions, where time is often limited and the changeover between trainees is close to immediate, lecture and tutorial formats are often used to deliver key clinical and logistical information regarding on-call work [23]. These can be delivered to larger groups, faster, without the need for intensive simulation resources and facilitators while still providing critical information. Nevertheless, Blamey et al*.* did highlight that didactic lectures on performing on-call tasks were inferior in comparison to a combination of an introductory tutorial and subsequent on-call simulation [7].

Furthermore, 3 publications explicitly supported the concept that on-call simulation during induction periods should be standard practice and helps improve confidence in trainees which may in turn improve their clinical performance [5, 20, 21]. This was particularly relevant in specialities where newly qualified doctors lacked clinical experience and medical school exposure as Blamey et al*.* demonstrated in their psychiatry specific on-call simulation [7]. In the current UK sphere of rotational training, where newly qualified doctors rotate through different specialities on a 4 monthly basis, a targeted specialty specific simulation of common tasks can serve as a vital adjunct to on-call confidence.

### On-call simulation remains resource intensive

The transition to virtual simulations was driven largely in part to the COVID-19 pandemic and the requirement for social distancing [7, 26]. Stone et al*.* argued that virtual formats provided a good substitute for in-situ on-call simulations [22]. Furthermore, the authors highlighted key benefits in the delivery of virtual simulations, including increased facilitator availability, the lack of requirement for physical simulation space and the greater scalability and accessibility of simulations to participants when conducted online [7, 22].

Nonetheless, despite the reported success of virtual models, Blamey et al*.* make the case that in-situ simulation remains the gold standard [7]. Primarily, as it reflects the psychological fidelity and reality of on-call work. It is difficult to evaluate the two techniques in the absence of direct comparative studies; however, there are certainly merits to both.

In the era of increasing demand on medical educators, including the expansion of medical school places [27], there remains an absolute need for novel innovation in this field in relation to scalability and accessibility. An alternative low-resource approach to virtual simulation could be the incorporation of tabletop methods into on-call simulation practice. Tabletop methodology is extensively used in medical disaster management where prioritisation and management of patients in simulated mass casualty events is practiced [11, 28]. It is often employed as a scalable low-resource activity that can involve multiple participants simultaneously. The focus of tabletop methodology on clinical decision making and prioritisation of tasks makes it translatable into on-call simulation while providing the crucial benefits of scalability and accessibility [10]. Tabletop on-call simulation would allow a high volume of participants to practice critical non-technical skills without the need for expensive high-fidelity equipment and high staffing requirements [8, 9]. On the other hand, this form of simulation may compromise the psychological realism of the experience, a factor that has been highlighted as crucial for good learning outcomes [25]. It is likely that tabletop simulation would find a role as a suitable adjunct rather than substituting traditional on-call simulation methodologies. However, it remains a key instrument in the facilitator’s repertoire to help augment and deliver an on-call simulation experience at scale. Currently there is a paucity of published, accessible tabletop on-call simulations and this is a key area of further development if were are to keep pace with the growing demand for medical education.

### Interprofessional education

Innovation in the design of on-call simulation is not restricted to the adaptation of tabletop methodology. Interprofessional Education (IPE) has gained increasing traction as educators attempt to more closely emulate the clinical environment and the multidisciplinary working that underpins effective patient care [29]. Interestingly, none of the reviewed on-call simulations incorporated IPE methods and the focus remained on improving learning outcomes amongst medical students and doctors. However, the design of on-call simulations lends itself well to introducing other healthcare roles, particularly nursing staff.

Ivarson et al*.* had previously demonstrated the merits of on-call IPE exercises in the context of interprofessional training wards (IPWTs) [30]. IPTWs are learning attachments situated in real clinical environments where students practice under the supervision of working clinicians. Ivarson et al*.* ‘Call the On-Call’ experience saw medical and nursing students collaborate in an out-of-hours scenario relating to either a real or simulated clinical scenario, with the nursing students escalating for clinical advice to their medical colleague [30]. This was supervised in situ by the on-call doctor and allocated ward nurse. The authors emphasised that the focus of this exercise remained on improving interprofessional communication in acute clinical situations rather than management of the scenario. Nevertheless, the publication highlighted that sessions provided all participants with experiential learning on effective multidisciplinary communication and helped provide insights into the roles of doctors and nurses in the clinical environment.

There is scope for such an exercise to extend beyond the aim of augmenting interprofessional communication and to focus on improving participants’ confidence in managing common on-call clinical scenarios. Traditional on-call simulation formats involve facilitators playing the role of nursing staff, escalating tasks to the on-call participant [8]. Adapting the Ivarson et al*.* IPWT model, facilitators could be replaced with nursing roles allowing nurses and students to practice profession specific competencies such as escalating deteriorating patients in a simulated environment while simultaneously retaining the advantages of enhanced interprofessional communication training [30, 31]. Although this would require a significant logistical undertaking to ensure tasks remain relevant and useful to both professions, the IPE element would help address the current dearth of educational provision in this area, augment simulation fidelity for all participants and foster the interprofessional collaboration that is critical to good patient outcomes [30–32].

## Conclusion

In conclusion the evidence suggests that on-call simulation improves self-reported confidence amongst participants in performing on-call tasks [8–10, 17]. However, it remains unclear if on-call simulation confers these benefits long-term [5]. Studies also fail to evidence, or neglect, establishing improved clinical performance. Further investigation, particularly in relation to the development of non-technical competencies, is needed to address this disparity.

The design of simulated tasks is critical to ensure effective delivery of simulation-based education reflective of the reality of on-call work [4]. Scenarios should challenge participants to mimic the cognitive load and psychological pressures of on-call simulation [25]. Stakeholder involvement guiding the creation of tasks can help improve realism and identify learning needs [3, 7].

Induction programmes should incorporate on-call simulation, particularly in specialties where participants often have limited previous exposure [7]. This fosters the development of key technical and non-technical skills and bridges the gap between theoretical knowledge and practical competence [22].

Notably, the resource-intensive nature of on-call simulation persists, even with the shift towards virtual settings and the integration of low-fidelity models [4, 5, 16]. As the field of medical education continues to expand investigating alternatives such as tabletop simulations [10, 11] should become a focus for innovation. Furthermore, the lack of integrated IPE throughout current on-call simulations highlights a key area for further development [30, 31]. On-call simulation exercises should be extended beyond the current cohorts of doctors and medical students to include and benefit other healthcare professionals.

### Supplementary Information

Supplementary Material 1.

## Data Availability

The datasets used and analysed during the current study are available from the corresponding author on reasonable request.

## Definitions

Newly qualified doctors: *Doctors in their first two years of clinical practice*
Foundation doctors/trainees: *UK Doctors enrolled in the foundation training programme, often their first two years of clinical practice*
Participants: *Individuals undertaking the simulation exercise*
OSAT: *Objective Simulation Assessment Tool*
ABCDE: *Airway, Breathing, Circulation, Disability, Exposure. A method for assessing acutely deteriorating patients*
IPE: *Interprofessional Education*
IPWT: *Interprofessional Training Wards*
