# Supplementary material for: Preparing clinicians for practice: effectiveness and design of on-call simulation
Source: BMC Med Educ. 2024 Jun 5;24:623. doi: 10.1186/s12909-024-05495-y (PMC11155049; doi:10.1186/s12909-024-05495-y)
Supplement: Supplementary file 1 — Supplementary Material 1. [file 12909_2024_5495_MOESM1_ESM.docx]

***Appendix One***

*MeSH terms and Keywords developed by specialist librarian for use in literature search*

| **MeSH terms and Keywords** |
| --- |
| Simulation Training |
| Simulat* |
| Experience |
| Exp* |
| On Call |
| On-Call |
| Bleep* |

***Appendix two***

Evidence search syntax

A review of literature surrounding existing bleep simulations. Lucy Wells. (18th August, 2023). BRIGHTON, UK: Sussex Health Knowledge and Libraries.

Numbers of search results in bold italics. Searches presented in numeric chronological order.

Ovid MEDLINE(R) ALL <1946 to August 16, 2023>

1. exp Simulation Training/ ***11683***
2. (simulat* or experience or on call or on-call).ti,ab. ***1504072***
3. 1 or 2 ***1506151***
4. bleep*.ti,ab. ***115***
5. 3 and 4 ***25***

Embase <1974 to 2023 Week 32>

1. exp simulation training/ or exp simulation/ ***454015***
2. (simulat* or experience or on call or on-call).ti,ab. ***1895149***
3. 1 or 2 ***2022610***
4. bleep*.ti,ab. ***360***
5. 3 and 4 ***116***

The datasets used and analysed during the current study are available from the corresponding author on reasonable request.

***Appendix Three***

*Study inclusion and exclusion criteria in table format.*

| **Inclusion** | **Exclusion** |
| --- | --- |
| Articles in any language | Articles not relating to the study/use of on-call simulation |
| Articles published between 1946 – Aug 2023 | Articles relating to single scenario simulations with no on-call simulation element |
| Published articles or abstracts relating to the study/use of on-call simulation | Articles reporting on preparatory resources for on-call work without inclusion of on-call simulation |
| On-call simulation studies in medical students and newly qualified doctor cohorts | Articles reviewing the use of on-call simulation in populations outside of medical students and newly qualified doctors. |
